# γδ T cells in diabetes mellitus: dual roles and therapeutic implications

**DOI:** 10.3389/fimmu.2025.1693466

**Published:** 2025-11-18

**Authors:** Bing Wang, Qi Li, Qiuyue Wang

**Affiliations:** Department of Endocrinology and Metabolism, The First Affiliated Hospital of China Medical University, Shenyang, Liaoning, China

**Keywords:** gamma delta T cells, cytokines, diabetes mellitus, autoimmunity, insulin resistance, inflammation, diabetic complications

## Abstract

Diabetes mellitus is primarily categorized into type 1 diabetes mellitus (T1DM) and type 2 diabetes mellitus (T2DM), which exhibit distinct pathogenic mechanisms. T1DM is characterized by an absolute deficiency of insulin secretion, predominantly resulting from the autoimmune-mediated destruction of pancreatic beta cells. In contrast, T2DM arises from a combination of insulin resistance in peripheral tissues and a compensatory insulin secretory response that ultimately becomes inadequate. The pathogenesis of diabetes mellitus is orchestrated through bidirectional crosstalk between autoimmune aggression and metabolic derangement. γδ T cells, innate-like lymphocytes bridging innate and adaptive immunity, play pivotal roles in tissue homeostasis, inflammation, and immunity through cytokine production and cytotoxicity. This review comprehensively examines the dual roles of γδ T cells across diabetes mellitus types. Furthermore, γδ T cells contribute to diabetic complications and are profoundly affected by the diabetic milieu, leading to defective anti-infection and anti-tumor immunity. We discuss emerging therapeutic strategies targeting γδ T cells or their effector pathways and highlight key knowledge gaps regarding subset-specific functions, dynamic changes during disease progression, and tissue-resident γδ T cell roles. Elucidating these mechanisms may provide a strong foundation for developing novel γδ T cell-based immunotherapies for diabetes mellitus and its complications.

## 1 Introduction

Diabetes mellitus (DM) is a chronic metabolic disorder characterized by persistent hyperglycemia. DM embodies a paradigm of autoimmune-metabolic crosstalk. Type 1 diabetes mellitus (T1DM) is defined by autoimmune destruction of pancreatic β-cells, while type 2 diabetes mellitus (T2DM) features metabolism-triggered autoinflammation. According to the 2021 Global Burden of Disease analysis, approximately 591 million individuals worldwide live with diabetes, a figure projected to reach 1.031 billion by 2050 (1). This escalating prevalence underscores DM’s status as a critical public health challenge. Suboptimal glycemic control predisposes patients to multi-system complications affecting ocular, renal, integumentary, and other organ systems (2, 3). Furthermore, DM significantly elevates mortality risks from infections and malignancies (4), with this systemic vulnerability linked to immune dysregulation. The innate immune system is involved in the pathogenesis of DM and its chronic complications (5). Within this context, γδ T cells emerge as unexplored arbiters of diabetic autoimmunity in T1DM and as drivers of obesity-related inflammation in T2DM. Serving as a bridge between innate and adaptive immunity (6), γδ T cells exert unique functions in tissue immunosurveillance and inflammatory modulation through major histocompatibility complex (MHC) -unrestricted activation and cytokine secretion, thereby regulating αβ T cells and other immune effectors (7). Notably, the diabetic milieu may reciprocally impair γδ T cell function.

As an endocrine-metabolic disease driven primarily by autoimmunity, γδ T cells may mediate either protective or destructive effects on pancreatic β-cells in T1DM. γδ T cells also critically interact with obesity-induced insulin resistance, which is the fundamental mechanism in T2DM development. Visceral adipose tissue (AT) in obese individuals shows marked γδ T cell expansion, accounting for over 95% of tissue-resident immune cells (8), highlighting their dominance in the adipose niche. IL-17, a key cytokine secreted by γδ T cells in AT (9, 10), suppresses glucose uptake in skeletal muscle and impairs insulin sensitivity in hepatocytes (11), positioning γδ T cells as drivers of obesity-related inflammation in T2DM. Additionally, γδ T cells participate in diabetic complications through epithelial repair mechanisms in lung and skin tissues (6), potentially influencing infection susceptibility and wound healing in DM. While γδ T cells exhibit context-dependent pro- or anti-inflammatory roles in DM, their subset-specific functions, temporal dynamics, and therapeutic targeting potential remain incompletely defined. Elucidating these mechanisms may provide a critical foundation for novel immunomodulatory strategies against DM.

## 2 Gamma delta T cell

### 2.1 Origin and development

γδ T cells are innate-like lymphocytes that fundamentally differ from conventional αβ T cells in developmental origin and activation mechanisms. T cell development broadly follows a sequential process. Initially, hematopoietic stem cells differentiate into lymphoid stem cells within the bone marrow hematopoietic inductive microenvironment. These lymphoid stem cells further develop into pro-T cells, which then migrate via the bloodstream to the thymus (12). Within the thymic microenvironment, pro-T cells differentiate sequentially through the double-negative (DN), double-positive (DP), and single-positive (SP) stages, ultimately maturing into functional T cells (13). The pro-T cell stage represents the branch point at which αβ T cells and γδ T cells begin to diverge into distinct lineages. During differentiation, bone marrow-derived progenitor T cells migrate to the thymus where they undergo TCR gene rearrangement at the pro-T cell stage. In the thymus, αβ T cells constitute over 95% of the total T cell population, whereas γδ T cells account for less than 5%. γδ T cells arise from DN thymocytes and undergo TCRγ and TCRδ rearrangement prior to TCRβ recombination, subsequently expressing the γδTCR/CD3 complex on their plasma membrane (14). Notably, rare thymocytes co-express both γδ and αβ TCRs (15). Their antigen recognition mechanisms differ significantly from those of αβ T cells. γδ T cells directly recognize diverse antigens in an MHC-independent manner. This non-classical recognition stems from unique TCR diversity generation mechanisms. Functionally, γδ T cells rapidly secrete cytokines or directly lyse target cells via the NKG2D**–**ligand pathway, playing pivotal roles in anti-pathogen defense, tumor immunosurveillance and tissue repair (16). The high conservation of TCR repertoires, tissue distribution, and functional subsets between human and murine γδ T cells establishes mice as essential model organisms for mechanistic studies.

### 2.2 Subsets

**Figure 1:**
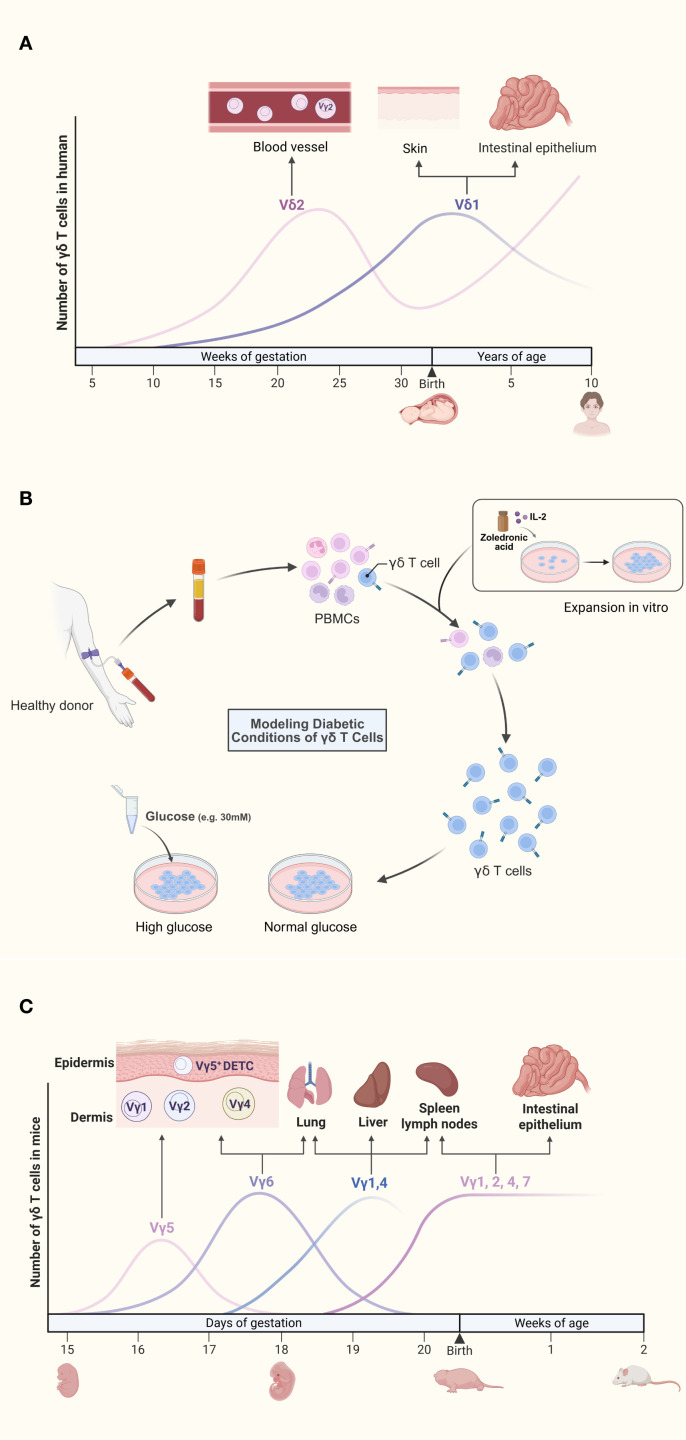
**(A)** Dynamic reconstitution of human γδ T cell subsets from fetal development to childhood. The earliest rearrangements involve the Vγ9 and Vδ2 gene segments. Although Vγ9Vδ2 T cells expand to form a major subset by mid-gestation, Vδ1 T cells are the dominant population in umbilical cord blood at birth. During postnatal development, the peripheral γδ T cell compartment undergoes a substantial reconstitution, characterized by a marked expansion of the Vγ9Vδ2 population, which becomes the predominant subset (>75% of circulating γδ T cells) by approximately 10 years of age. **(B)** Isolation, *in vitro* expansion, and high-glucose treatment of γδ T cells from diabetic models. Peripheral blood mononuclear cells (PBMCs) are isolated from peripheral venous blood via density-gradient centrifugation. Cultures are stimulated with zoledronate and recombinant human IL-2 to promote γδ T cell activation and proliferation. After 10–14 days of expansion, γδ T cells display robust proliferation, whereas other immune cell subsets progressively undergo apoptosis. Finally, the expanded γδ T cells are exposed to high-glucose conditions to mimic the diabetic microenvironment and assess their functional responses. **(C)**. Spatiotemporal ontogeny and tissue distribution of murine γδ T cell subsets. During embryogenesis, the first wave of γδ T cells expresses monoclonal Vγ5Vδ1 TCRs, which migrate to and establish permanent residency within the epidermis. Subsequent Vγ6^+^ subsets traffic to the dermis, peritoneal cavity, and adipose tissue, while Vγ4^+^ subsets emerge concurrently and localize to the lung, skin dermis, and lymph nodes. In the perinatal period, Vγ7^+^ subsets colonize the intestinal tract, whereas polyclonal Vγ1^+^ and Vγ4^+^ subsets distribute broadly across peripheral lymphoid organs.

Human γδ T cells are classified into Vδ1, Vδ2, and Vδ3 subsets based on δ chain usage. From embryonic stages to childhood, the relative frequencies of the two primary human γδ T cell subsets (Vδ1 and Vδ2) undergo dynamic shifts (**Figure 1A**). The earliest rearrangements in the gamma/delta T cell lineage involve the Vγ9 and Vδ2 gene segments. Evidence of this process appears in the fetal liver during gestational 5–6 weeks and in the fetal thymus from the 8th week onward (17). Following this early development, Vγ9Vδ2^+^ T cells expand to constitute the majority of the gamma/delta repertoire by midgestation (20–30 weeks) (17). From birth to approximately 10 years of age, the peripheral γδ T cell compartment undergoes substantial maturation, marked not only by an increase in total numbers but also by a dramatic reconstitution of its subset composition, wherein the Vγ9Vδ2 population expands from a small fraction to a majority (>75% circulating γδ T cells) (18). In contrast to their minority status in adults, Vδ1 T cells represent the dominant γδ T cell subset in umbilical cord blood at birth (18). Vδ1 T cells, which account for only a minority in peripheral blood, are enriched in barrier tissues such as the skin and mucosa-associated lymphoid tissue (MALT), and recognize antigens presented by CD1 molecules; Vδ2 T cells, which dominate in peripheral blood and lymphoid organs, are primarily recognize phosphoantigens derived from microbial metabolism (e.g., HMBPP) (6, 19, 20). Zoledronate is a bisphosphonate. Vδ1 T cells are non-responsive to bisphosphonates or phosphoantigens. Culturing γδ T cells under high-glucose conditions requires an initial *in vitro* expansion step (**Figure 1B**). The established method of expanding Vδ2 T cells *in vitro* with zoledronate and IL-2 has made this subset a major subject of study in diabetic autoimmunity research (21). This subset-specific distribution and functional specialization underscore the need to avoid overgeneralization when studying γδ T cells in diabetic complications.

Murine γδ T cell subsets are primarily classified according to Vγ chain usage. Developmentally, γδ T cells represent the earliest T cell population emerging in the embryonic mouse thymus. During embryogenesis, the first wave expresses monoclonal Vγ5Vδ1 TCRs that home to and permanently reside in the skin epidermis (17). Subsequently, additional subsets develop and localize to specific niches (**Figure 1C**). The skin epidermis harbors a specialized subset termed dendritic epidermal T cells (DETC), originating from embryonic Vγ3Vδ1 precursors (22). Postnatally, polyclonal CD27^+^ Vγ1 and CD27^+^ Vγ4 subsets mature predominantly in the liver and lymph nodes (16). Functional specialization of murine γδ T cells is governed by Vγ chains, establishing mice as essential experimental models (17, 23). Despite conserved tissue distribution and phenotypic functions between human and murine γδ T cells, no strict subset equivalency exists. Researchers must judiciously select models and subsets based on target tissue microenvironments and specific biological questions (**Table 1**).

**Table 1:** Human and mice γδ T cell subsets: tissue localization and dominant cytokine profiles.

Human	Mice	Major tissue distribution	Predominant cytokine	Reference
Vδ1	Vγ6	Mucosal tissue/Lung	IL-17	(16, 17, 20, 24–29)
	Vγ7	Intestinal epithelium	IFN-γ, TNF-α	
	Vγ5^+^ (DETC)	Epidermis	IFN-γ, TNF-α,	
	Vγ1	Lamina propria of the intestine	IFN-γ	
	Vγ1	Inflammatory tissue/Brain(Traumatic brain injury)	IL-10, TGF-β	
Vδ2 (Vγ9Vδ2)	Vγ1	Spleen/Lymph nodes/Liver	IFN-γ	(17, 19, 20)
	Vγ4	Peripheral blood/Liver/Secondary lymphoid organs	IFN-γ/TNF-α/IL-17	
Vδ3^+^ (Rare)	–	Liver/Intestine	IL-17/IFN-γ	(17, 20, 30)

This table summarizes the predominant subsets, their major tissue localization, and key effector cytokines based on the literature. DETC, dendritic epidermal T cells. Cytokines listed (e.g., IFN-γ/IL-17) indicate the predominant ones produced by the subset, with multiple cytokines indicating potential co-production or context-dependent expression. The murine Vγ nomenclature is provided as the functional counterpart to the human subsets where applicable.

Beyond the aforementioned major subtype classification based on TCR chains, γδ T cells can also be categorized into distinct subsets according to their cluster of differentiation (CD) profiles. Fundamentally, the T-cell receptor (TCR) complex consists of receptor subunits (either TCRαβ or TCRγδ) and the associated CD3 subunits (CD3γ, δ, ϵ, and ζ) (31). Consequently, like all mature T cells, γδ T cells uniformly express the CD3 complex. γδ T cells predominantly exhibit a CD4^-^CD8^-^ double-negative phenotype, with a minor subset expressing CD8^+^ (32). A single-cell RNA sequencing study in NOD mice revealed an abnormal expansion of double-negative T cells (33), although the specific role of γδ T cells within this population requires further investigation. Furthermore, Different functional γδ cell subsets can be classified by CD27. Mature TCRαβ^+^ thymocytes homogeneously express CD27, while γδ T cells represent only a small subset of the CD27^+^ thymocyte population. Human Vδ2 T cells can be functionally subdivided into naïve (CD45RA^+^CD27^+^), central memory (CD45RA^-^CD27^+^), effector memory (CD45RA^-^CD27^-^), and terminally differentiated effector (often CD45RA^-^CD27^-^ or other combinations) phenotypes (17). CD27^-^ thymocytes (approximately 10% of all γδ thymocytes) preferentially differentiate into IL-17A–secreting cells and CD27^+^ subsets primarily generate IFN-γ (34).

### 2.3 Effector functions

γδ T cells can be activated by specific cytokines to produce effector cytokines. While essential for tissue homeostasis at physiological levels, excessive concentrations of pro-inflammatory cytokines, such as TNF-α and IL-17, drive chronic inflammation (6). IFN-γ production requires synergistic IL-12 and IL-18 signaling, and IL-17A secretion is induced by IL-23 and IL-1β (19). Notably, single cytokine stimulation fails to elicit robust responses (24). Although Th17 cells are primary IL-17A producers, γδ T cells serve as significant contributors, particularly during early mucosal immune defense. Murine γδ T cell development critically depends on IL-7 and IL-15 (35). IL-15 and IL-2 drive IFN-γ^+^ subsets (Vγ5^+^ DETC, Vγ7^+^, Vγ1^+^), and IL-7 promotes IL-17A^+^ γδ T cells (predominantly CD27^-^Vγ6^+^) (17) (25).

**Figure 2:**
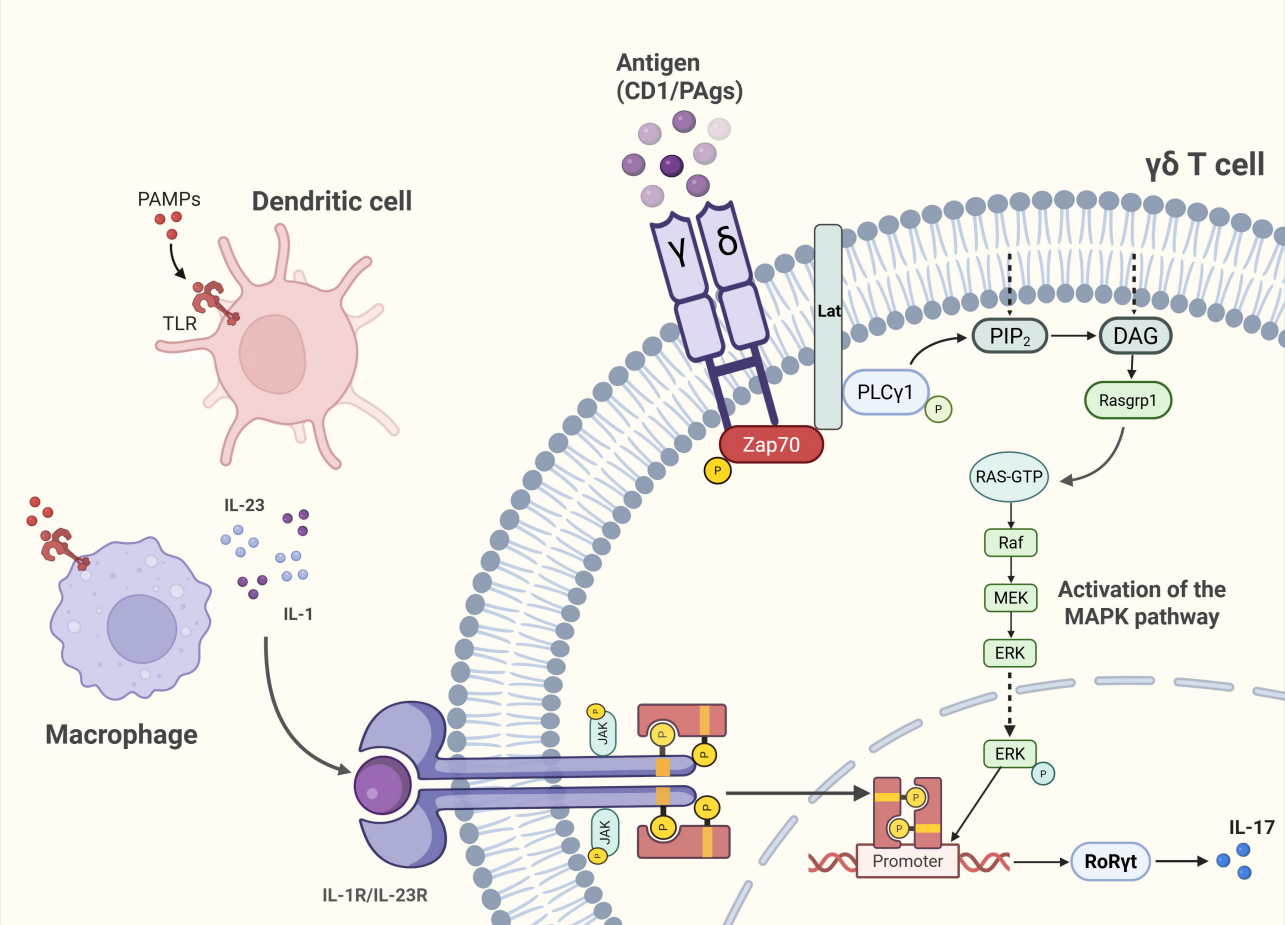
Unique activation pathways of γδ T cells. Pathogen-sensing route (TCR-independent): Engagement of PAMPs with PRRs on dendritic cells (DCs) and macrophages induces IL-23 and IL-1β release, directly stimulating γδ T cells to secrete IL-17A without TCR involvement. TCR-dependent route: Antigen recognition via the γδ TCR activates the ERK/MAPK signaling cascade, driving transcriptional polarization toward RORγt in IL-17–producing subsets.

γδ T cells have several activation pathways (**Figure 2**). The JAK2/STAT3/RORγt axis implicated in inflammatory and fibrotic diseases operates in immune cells (36–38). However, previous studies on this pathway have mostly focused on Th17 cells, with relatively few studies on γδ T cells that secrete IL-17. When pathogen-associated molecular patterns (PAMP) bind to pathogen recognition receptors(PRRs), DCs or macrophages release IL-23 and IL-1β directly triggers γδ T cell IL-17A secretion without TCR involvement (39). In addition, the TCR signaling pathway can also regulate the secretion of cytokines by γδ T cells. According to some research findings, γδ T cells may utilize molecular mechanisms during TCR signaling activation that differ from those of αβ T cells (40, 41). Although this pathway has not been fully elucidated, the signaling mechanisms of αβ TCR are considered largely similar to those of γδ TCR (14). Therefore, the γδ TCR signaling pathway can be understood within the framework established for αβ TCR signaling. Kinases act as critical drivers of TCR signaling. ZAP-70, a member of the Syk family kinases, is recruited to the TCR complex and the transmembrane adaptor protein LAT upon TCR activation, where it undergoes phosphorylation (42, 43). Phosphorylated LAT then provides docking sites for signaling enzymes such as PLCγ1. Subsequently, PLCγ1 hydrolyzes phosphatidylinositol-4,5-bisphosphate (PIP_2_) into inositol-1,4,5-trisphosphate (IP_3_) and diacylglycerol (DAG). DAG recruits Ras guanine nucleotide-releasing protein 1 (RasGRP1) to the plasma membrane, leading to activation of the Ras-ERK pathway (14). In γδ T cells, the main transcription factors regulating the expression of IL-17 or IFN-γ downstream of the TCR signaling pathway are RORγt or Tbx21(T-bet), which are partially transduced through the extracellular-signal related kinases/mitogen-activated protein kinases (ERK/MAPK) pathway (25).Functionally constrained by their developmental programming, γδ T cell effector fates remain largely unaltered in response to exogenous cytokines. Physiological cytokine secretion maintains tissue homeostasis, whereas pathological overproduction contributes to skin inflammation, atopic dermatitis, and autoimmune arthritis (44, 45). Therapeutic IL-17 blockade attenuates such inflammation (46), highlighting γδ T cell subset modulation as a promising, though still investigational, intervention strategy.

## 3 Gamma delta T cells in diabetes mellitus pathogenesis

### 3.1 The dual role and therapeutic potential of γδ T cells in T1DM

Several clinical studies suggest a potential association between γδ T cells and T1DM. Newly diagnosed T1DM patients exhibit a reduced mean proportion of peripheral blood γδ T cells, with further depletion observed after one year of insulin therapy (47). In islet cell antibody (ICA)-positive relatives of T1DM probands, who represent a high-risk group for future diabetes development, a high percentage of γδ T cells is associated with ICA positivity (48, 49), potentially reflecting stage-specific immune alterations. In T1DM animal models, distinct γδ T cell subsets have been specifically investigated. The non-obese diabetic (NOD) mouse serves as an ideal model for studying the immune basis and treatment of T1DM (50). Research indicates that Vγ4^+^ γδ T cells in NOD mice can suppress T1DM development by producing IL-17 and facilitating the differentiation of regulatory CD4^+^ αβ T cells in pancreatic lymph nodes; Vγ1^+^ cells, biased toward IFN-γ production, thereby promote a pro-inflammatory microenvironment conducive to T1DM pathogenesis (51). Within NOD mouse islets, the majority of infiltrating γδ T cells are IL-17-producing CD27^-^ cells, while the IFN-γ-producing subset expresses CD27 (34, 52). Although the proportion of peripheral γδ T cells increases in NOD mice, predominantly IL-17-producing cells, they do not exacerbate diabetes; instead, they confer protection by upregulating TGF-β production (53). A gluten-free diet enriches splenic naïve CD27^+^ γδ T cells in mice, potentially reducing type 1 diabetes susceptibility by preventing their differentiation into pro-autoreactive effector cells (54). Collectively, these findings demonstrate a dual role for γδ T cells in T1DM pathogenesis, primarily governed by their effector cytokine profile. IL-17-producing γδ T cells appear primarily protective, suppressing islet inflammation. In contrast, the IFN-γ-producing subset promotes disease progression. Notably, γδ T cells coordinate with αβ T cells to drive T1DM development but are insufficient to independently cause disease (52). The role of IL-17 remains particularly challenging to define, as studies using IL-17 blockade have reported either protective effects or no significant impact, while in diabetic complications it demonstrates a dual nature (55). These discrepancies appear to depend on the experimental animal model employed, the timing of intervention, and the specific cytokine microenvironment, highlighting the need for further investigation in future studies.

Insulin therapy remains a cornerstone treatment for T1DM and has been shown to possess immunomodulatory properties. T1DM patients show a significant increase in peripheral blood CD8^+^γδ T cells after 3–6 months of insulin treatment (56), suggesting the expansion of a potential regulatory subset induced by exogenous insulin. This concept is strongly supported by animals studies in which mucosal insulin administration promotes immune tolerance. Aerosol insulin induces autoimmune tolerance mediated by regulatory CD8^+^ γδ T cells, preventing T1DM in mice (57). Similarly, naso-respiratory insulin administration in NOD mice increases IL-10-producing CD8^+^ γδ T cells in pancreatic lymph nodes (58). The underlying mechanism may involve the unique antigen-recognition capability of γδ T cells. The TCRs of NOD mouse γδ T cells exhibit specific reactivity to multiple insulin antigens, likely through an APC-independent mechanism (59), indicating TCR-dependent recognition. For instance, γδ TCRs can recognize the insulin oxidized B:9–23 peptide, naturally generated during insulin degradation in β cells, which contains the essential Cys19 residue for γδ T cell responses (60). Based on these findings, a mechanistic model can be proposed. Mucosal insulin administration may enhance the local presentation or availability of insulin-derived peptides (such as B:9–23) in respiratory mucosa. This setting likely promotes the engagement of insulin-reactive γδ T cell TCRs, leading to their activation and functional polarization. These activated CD8^+^ γδ T cells acquire a regulatory phenotype, characterized by secretion of the potent anti-inflammatory cytokine IL-10. These cells subsequently migrate to pancreatic lymph nodes, where local IL-10 production may suppress the activation and effector functions of autoreactive αβ T cells (61), thereby reestablishing immune tolerance and preventing β cell destruction. Collectively, these studies demonstrate that insulin, beyond its metabolic role, may exert immunomodulatory effects that counter T1DM pathogenesis. γδ T cells may modulate immune function through potential responses to insulin antigens, potentially influencing T1DM onset and progression.

The role of γδ T cells in T1DM is gradually being elucidated (**Table 2**). However, current human studies present limitations. Firstly, clinical assessments predominantly measure total γδ T cells without distinguishing functionally heterogeneous subsets (Vδ1/Vδ2), hindering the precise identification of protective versus pathogenic subsets and contributing to observed discrepancies. Defining the γδ T cell subset repertoire in human T1DM patients remains a future objective. Additionally, the lack of phenotypic and functional tracking of islet-resident γδ T cells in humans impedes understanding of their direct role in islet autoimmunity. The inherent difficulty in obtaining pancreatic tissue samples from T1DM patients, who rarely undergo surgical intervention, presents a major obstacle. Currently, there is experience in procuring pancreatic tissue from individuals with T1DM. Pancreatic tissue samples from donors with T1DM can be obtained through nPOD (https://npod.org). Studies utilizing laparoscopic pancreatic biopsy have revealed immunological changes in the islets of newly diagnosed T1DM patients, without reporting major complications (62). However, the Diabetes Virus Detection Study (DiViD) collected larger pancreatic tissue samples via caudal pancreatectomy from adults recently diagnosed with T1DM, which resulted in some patients experiencing postoperative bleeding and leakage of amylase-rich pancreatic juice (63). Consequently, it was deemed unethical to continue the study. Significant challenges remain in the acquisition of pancreatic tissue from patients with T1DM.

**Table 2:** The role of γδ T cells in T1DM.

Study type	Cell type/feature	Dual function/role	Possible mechanisms	Reference
Clinical Studies	Total peripheral blood γδ T cells	↓ (Decreased in newly diagnosed T1DM patients)	Autoimmune progression	(47)
		↓ (Further depleted after 1 year of insulin therapy)		
		↑ (Correlated with ICA positivity)	Immune dysregulation state	(48)
	Peripheral blood CD8^+^ γδ T cells	↑ (Significantly increased in T1DM patients post-insulin therapy)	Immunomodulatory effect	(56)
Animal Model (NOD mice)	Vγ4^+^/IL-17-produing γδ T cells	Protective role (predominantly): Suppresses T1DM development	1. Secretion of IL-17 2. Promotion of regulatory CD4^+^ αβ T cell differentiation 3. Upregulation of TGF-β	(34, 51, 52)
	Vγ1^+^/CD27^-^γδ T cells	Pathogenic role: Promotes T1DM development	1.Secretion of IFN-γ 2.Secretion of IL-17	
	CD8^+^ γδ T cells in pancreatic lymph nodes (Post naso-respiratory insulin)	↑	Secretion of IL-10	(57, 58)
	γδ T cells response to insulin antigens	Insulin modulates immune responses via γδ T cells, influencing T1DM onset/progression	1. TCR-dependent recognition of insulin antigens 2. Recognition of insulin oxidized B:9–23 peptide (Cys19-dependent) 3. APC-independent mechanism	(59, 60)

This table summarizes the findings from clinical and animal model (NOD mice) studies on the frequency, function, and mechanisms of distinct γδ T-cell subsets in T1DM. Arrows (↑ increase, ↓ decrease) indicate significant changes in cell frequency or function associated with disease state or treatment. The proposed mechanisms are derived from the referenced studies. APC, antigen-presenting cell; ICA, islet cell antibody; NOD, non-obese diabetic; TCR, T-cell receptor; TGF-β, transforming growth factor beta.

### 3.2 γδ T cells in T2DM: balancing systemic exhaustion and tissue inflammation

Studies have shown that patients with T2DM exhibit increased monocyte counts, reflecting exacerbated chronic inflammation and immune activation, which promotes insulin resistance through the secretion of pro-inflammatory cytokines (64). Insulin resistance (IR), a central feature of obesity-related metabolic dysregulation, manifests as reduced insulin responsiveness in adipose, hepatic, and muscle tissues, ultimately leading to β-cell failure and T2DM onset. Obesity-induced chronic low-grade inflammation is a key driver of IR. The chronic inflammatory process is embedded within an immune-mediated proinflammatory environment, wherein γδ T cells exhibit complex immunoregulatory roles. First, both obese individuals and obese T2DM patients display significant dysfunction in the peripheral Vγ9Vδ2 T cell subset, characterized by diminished IFN-γ secretion (65, 66). In obese individuals, the proportion of Vγ9Vδ2 T cells in late apoptosis (Annexin V^+^ PI^+^) is significantly higher compared to those in early apoptosis (Annexin V^+^ PI^-^) (65). This accelerated apoptotic process in Vγ9Vδ2 T cells under obese conditions represents a potential mechanism contributing to their depletion in obese individuals. In addition, IL-2 stimulation can reverse this IFN-γ secretory defect, suggesting reversible functional suppression (65). Furthermore, the study indicated that obesity does not impair the capacity of Vγ9Vδ2 T cells to produce IFN-γ upon strong HDMAPP stimulation. This observation may also partially explain the improved fasting glucose, HbA1c, and insulin sensitivity observed in postmenopausal women with prediabetes and osteopenia following alendronate treatment (67). Bisphosphonates may restore the capacity of Vγ9Vδ2 T cells to produce cytokines such as IFN-γ through their activation. Consequently, restoring peripheral γδ T cell function represents a promising therapeutic avenue for T2DM. Within peripheral blood mononuclear cells (PBMCs) of T2DM patients, γδ T cells demonstrate increased cytotoxicity and expansion (68). In contrast to their systemic exhaustion, γδ T cells within adipose tissue (AT) exhibit a pro-inflammatory, tissue-resident phenotype. In AT of mice, two major γδ T cell populations exist: a CD3^ϵlow^CD27^+^ subset secreting IFN-γ, and a CD3^ϵhigh^CD27^-^ subset producing IL-17A and TNF-α (69). Adipose-resident γδ T cells serve as the primary source of IL-17A in adipose tissue. These IL-17-producing γδ T cells exhibit robust diurnal rhythms in RORγt and IL-17A expression, playing a critical role in systemic metabolic homeostasis by sustaining *de novo* lipogenesis (DNL) (70). Dysregulation of DNL is associated with metabolic disorders such as obesity and type 2 diabetes (71).The proportion of γδ T cells increases in the livers of individuals with non-alcoholic steatohepatiti (72). Paradoxically, clinical studies report significantly lower serum IL-17A levels in T2DM patients compared to normoglycemic controls (73), indicating a potential dissociation between local tissue inflammation and systemic immune responses, reflecting compartmentalized inflammation. This may arise from systemic immune exhaustion in diabetes reducing serum IL-17, while persistent activation of γδ T cells within the local adipose microenvironment elevates IL-17. This dichotomy may be explained by chronic metabolic insults which globally dampen immune responsiveness, leading to reduced cytokine output in circulation. Conversely, within specific niches like inflamed adipose tissue, local pro-inflammatory cytokines provide potent, compartmentalized signals that drive IL-17 production from resident γδ T cells. Furthermore, in obese mice, the predominant γδ T cell subsets accumulating in epididymal AT (eAT) are Vγ4^+^ and Vγ6^+^ T cells, which promote eAT inflammation by inhibiting the accumulation of anti-inflammatory M2 macrophages (74). M1 macrophages, conversely, enhance adipocyte inflammation and reduce insulin sensitivity via TNF-α production (75). High-fat (HF) diet-fed TCRδ^-/-^ mice exhibit reduced M1 macrophage accumulation and improved glucose clearance and insulin sensitivity post-insulin injection compared to TCRδ^+/+^ mice (74), suggesting adipose-resident γδ T cells promote insulin resistance.

NR4A nuclear receptors regulate hepatic gluconeogenesis and maintain inflammatory balance (76, 77). Dysregulated hepatic gluconeogenesis significantly impacts T2DM. NR4A1 and NR4A3 enhance insulin sensitivity in skeletal muscle and liver, yet are underexpressed in these tissues across various insulin-resistant animal models (78). In eAT, the abundance of γδ T cells decreased in mice fed with HF diet (79). In 3T3-L1 adipocytes, NR4A3 overexpression enhances insulin-stimulated glucose transport activity, potentially by increasing GLUT4 translocation to the plasma membrane or augmenting insulin-mediated IRS1 tyrosine phosphorylation and Akt phosphorylation (78). However, these studies primarily focus on NR4A in adipocytes or tissues. Research in cervical cancer cells indicates that transcription factors NR4A2/3 promote Vγ9Vδ2 T cell exhaustion (80), suggesting NR4A may regulate γδ T cells and influence immune-mediated inflammatory process in insulin resistance. Reduced adipocyte NR4A exacerbates insulin resistance, while diminished NR4A in adipose γδ T cells might delay their exhaustion, potentially contributing to sustained pro-inflammatory cytokine secretion and establishing a vicious cycle of metabolic and autoimmune inflammation. While NR4A receptors are implicated in metabolism and inflammation, their direct role in regulating γδ T cell function within the context of diabetic insulin resistance remains speculative and warrants dedicated investigation. If validated, NR4A could emerge as a key therapeutic target in T2DM. However, given divergent alterations in peripheral blood versus adipose tissue γδ T cells, NR4A expression may also exhibit opposing patterns. Specific investigations into NR4A family members within γδ T cells under diabetic conditions remain limited and warrant further study.

The JAK2/STAT3/RORγt pathway in γδ T cells may also contribute to T2DM pathogenesis. Enhanced expression of IL-23, JAK2, STAT3, and RORγt is observed in PBMCs of T2DM patients (81). RORγt is the key transcription factor for γδ T cell differentiation into IL-17–producing subsets, and this pathway is critically involved in Th17-mediated inflammation. Compared to non-diabetic or insulin-deficient islets, insulin-sufficient islets demonstrate elevated IL-17 expression in both β-cells and α-cells, though CD45^+^ cells are not the primary source of this IL-17 (82). In diabetic tissues, IL-17 contributes to impaired insulin signaling and β-cell dysfunction by activating the JNK pathway, promoting neutrophil infiltration into islets, and enhancing the expression of inflammatory cytokines and chemokines (83). Thus, investigating the specific molecular mechanisms underlying IL-17 production by CD45^-^ γδ T cells in T2DM islets represents a promising area for future research. γδ T cells might amplify AT inflammation and accelerate IR progression via this pathway, although the precise mechanisms require elucidation.

In summary, the role of γδ T cells in T2D is marked by a critical functional compartmentalization: systemic exhaustion in the periphery—evidenced by reduced Vγ9Vδ2 T cell frequency and impaired IFN-γ production—coexists with pro-inflammatory activation within metabolic tissues. In AT, resident γδ T cells promote insulin resistance through secretion of IL-17 and TNF-α, as well as by inducing pro-inflammatory macrophage polarization. This dichotomy resolves the apparent paradox of low serum IL-17 levels alongside localized tissue inflammation. The NR4A receptor family and the JAK2/STAT3/RORγt pathway have emerged as key potential mechanisms linking metabolic dysregulation to γδ T cell–driven inflammation, thereby providing an integrated model of their dual role in T2D pathogenesis (**Table 3**).

**Table 3:** Compartmentalized roles of γδ T cells in type 2 diabetes.

Compartment	Phenotype & function	Synthesized view	Reference
Peripheral Blood	↓ Vγ9Vδ2 T cell frequency ↓ IFN-γ production (exhaustion) ↑ Cytotoxic potential	Systemic Immune Dysregulation/Exhaustion	(65, 66)
Adipose Tissue (AT)	↑ γδ T cell infiltration ↑ IL-17A/TNF-α production Promotes M1 macrophage polarization	Local Pro-inflammatory Driver of Insulin Resistance	(69, 74, 75)
Key Discrepancy	Low Serum IL-17A vs. High Local IL-17 in tissues	Compartmentalized Inflammation: Systemic levels do not reflect pathogenic local tissue activity	–
Potential Regulators	NR4A receptors: Link metabolism and γδ T cell exhaustion. JAK2/STAT3/RORγt: Drives IL-17 production potential. (↑ JAK2/STAT3/RORγt pathway in PBMCs of T2D patients.)	These pathways may provide a molecular basis for the dysregulated γδ T cell responses.	(78–81)

This table is organized to clarify the dichotomous role of γδ T cells in T2D, contrasting their exhausted state in circulation with their pro-inflammatory activation in adipose tissue. The “Synthesized View” provides an integrated model that reconciles these observations, while arrows (↓, decrease; ↑, increase) indicate the direction of changes reported in studies.

### 3.3 Gestational state and gestational diabetes mellitus

Pregnancy represents a unique physiological state where γδ T cells contribute to localized immune responses. In healthy pregnant women, γδ T cells account for up to 50% of CD3^+^ T cells in the uterus. The majority of γδ T cells at the maternal-fetal interface (MFI) express Vδ1 and produce elevated levels of TGF-β and IL-10 (84). During early normal pregnancy, the Vδ1 subset at the MFI increases significantly (85), and exhibits fluctuations under progesterone regulation (86), highlighting its hormone-responsive functionality. Gestational diabetes mellitus (GDM), a common complication in pregnancy, prompts interest in the relationship between γδ T cells and GDM. The exploration of γδ T cells in GDM reveals alterations but lacks mechanistic clarity. Studies indicate alterations in lymphocyte subsets in GDM mothers and their newborns compared to health pregnancies. GDM mothers exhibit higher γδ T cell levels than healthy pregnant controls (87, 88). Specifically, GDM patients show increased peripheral blood total lymphocytes and CD8^+^ γδ T cells compared to normal glucose tolerance (NGT) controls, and GDM newborns have a higher proportion of CD8^+^ γδ T cell numbers than NGT newborns (89).

While current research has not yet directly elucidated the mechanistic pathways by which γδ T cells contribute to the pathogenesis of GDM, their known biological characteristics and the pathophysiology of GDM suggest several promising future research directions. First, given that progesterone regulates fluctuations in γδ T cells, the pronounced hormonal disturbances in GDM may disrupt the precise hormonal control of endometrial Vδ1 cells. This dysregulation could impair their production of cytokines such as IL-10 and TGF-β, thereby disturbing the immune-tolerant environment at the maternal-fetal interface and triggering local inflammation. Second, a shift of γδ T cells toward a pro-inflammatory profile may exacerbate insulin resistance. Approximately 80% of GDM cases arise from β-cell dysfunction against a background of chronic insulin resistance, a pathophysiology similar to that of T2DM (90). As significant producers of cytokines such as IFN-γ and IL-17, γδ T cells in GDM may undergo an abnormal shift toward such pro-inflammatory subsets, amplifying systemic and placental inflammation. This, in turn, can disrupt metabolic regulation via cytokine-mediated mechanisms and worsen insulin resistance in both maternal and fetal tissues. Finally, the observation that about 70% of prior GDM patients later develop T2DM (91) suggests the potential persistence of metabolic and immune dysregulation. The alterations in γδ T cells observed during GDM pregnancy may not be transient but rather represent a lasting immunological imprint. These long-lived, tissue-resident Vδ1 cells could sustain a low-grade inflammatory state, partially explaining the immunological link between GDM and subsequent T2DM. Future studies should directly analyze the functional status, subset distribution, and specific cytokine profiles of γδ T cells at the maternal-fetal interface and in the circulation of GDM patients to validate these hypotheses.

## 4 Diabetes-induced γδ T cells dysfunction

Clinical diabetes often involves pathological states like hyperglycemia or obesity. While γδ T cells contribute to diabetes pathogenesis, the diabetic milieu also impacts γδ T cells, inducing functional impairments that heighten susceptibility to infections and cancer in diabetic patients.

### 4.1 Anti-infection defects

γδ T cells constitute a crucial first line of defense against infections, acting early before primary αβ T cell responses develop. Vγ9Vδ2 T cells induce potent anti-infective effects by producing IFN-γ and lysing infected target cells (e.g., influenza, *Mycobacterium tuberculosis*) *(*65). T2DM patients are frequently overweight or obese. Obesity is associated with reduced peripheral Vγ9Vδ2 T cell numbers and weakened IFN-γ–dependent antiviral responses (65), potentially compromising anti-infective γδ T cell function in obese diabetics. Furthermore, hyperglycemia negatively impacts innate autoimmunity via oxidative stress induction and reduced cytokine production, increasing infection risk in DM (92–94). The lifetime risk of progressing from *Mycobacterium tuberculosis* infection to active tuberculosis (TB) significantly increases with immunosuppressive triggers like diabetes (95). Individuals with latent TB infection (LTBI) who have diabetes or prediabetes show reduced γδ T cells in PBMCs (96), potentially linked to diminished immune protection in LTBI. IL-17 is considered vital for anti-infective defense. γδ T cells secrete IL-17 early during mucosal surface infections, contributing to antibacterial immunity (39). Murine models of various pathogens (*S. pneumoniae, S. aureus, Escherichia coli*, influenza) demonstrate that γδ T cells can mobilize neutrophils via IL-17 secretion to combat infection (97–100). The IL-17-producing CD27^-^ γδ T cell subset rapidly expands during acute infection (34). γδ T cells and αβ-γδ T cells are also significant sources of IL-17 in early *S. aureus* infection and experimental autoimmune encephalomyelitis (EAE) (101, 102). Hyperglycemia in DM may impair IL-17 secretion, thereby increasing infection risk.

### 4.2 Antitumor impairment

**Figure 3:**
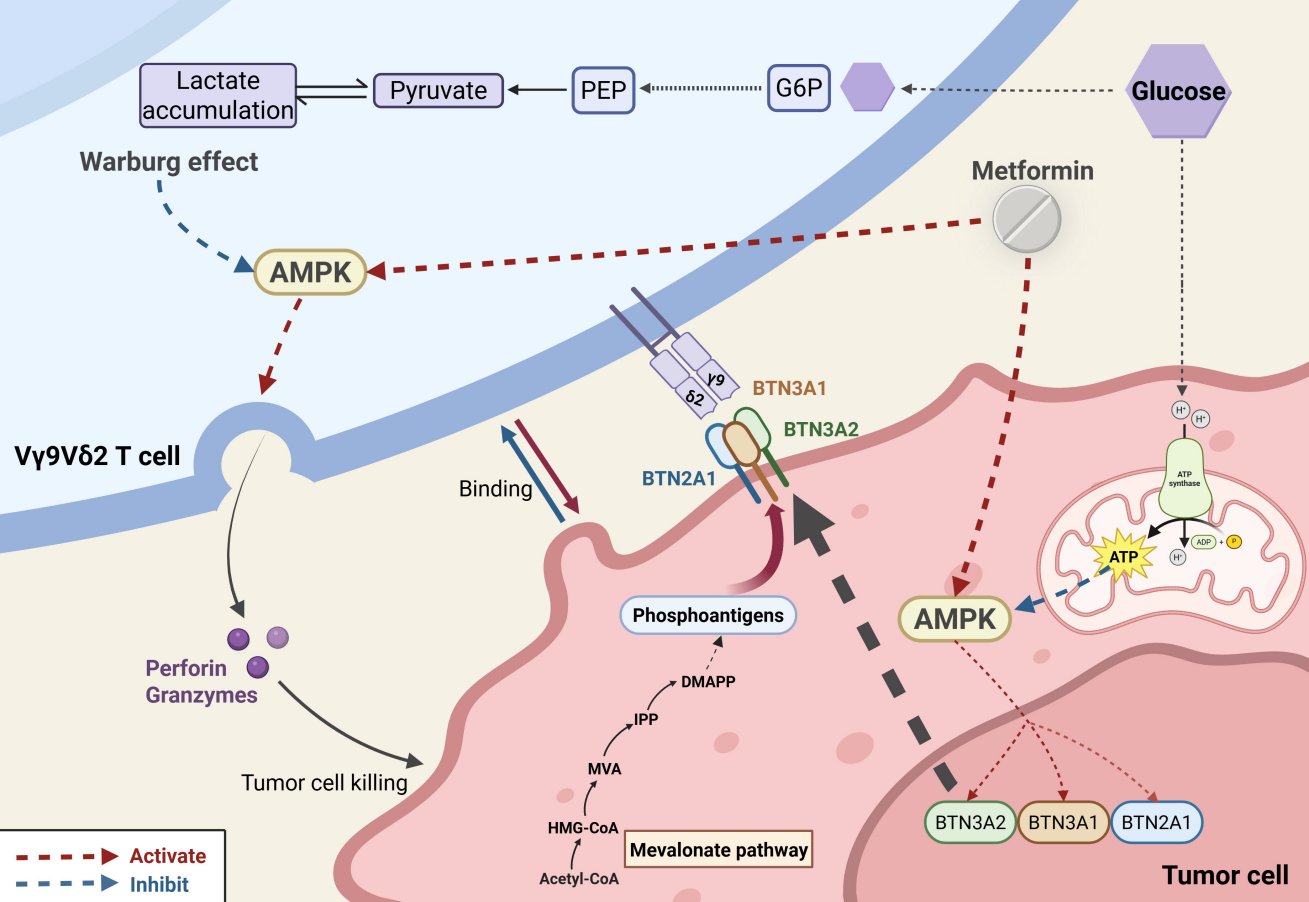
Metabolic–immunological interplay in hyperglycemia-driven tumor immunity dysfunction. Hyperglycemia impairs tumor immunity through two parallel mechanisms: (1) In γδ T cells, it induces a Warburg-like metabolic shift that suppresses AMPK activity, thereby disrupting lytic granule trafficking and reducing cytotoxicity. (2) In tumor cells, it downregulates AMPK-dependent expression of BTN2A1/BTN3A1 complexes, attenuating Vγ9Vδ2 TCR–mediated tumor recognition. Metformin reverses these defects via dual AMPK activation, restoring γδ T cell cytotoxicity and enhancing BTN2A1/BTN3A1 presentation on tumor cells.

γδ T cells play indispensable roles in antitumor immunity. This section focuses on how hyperglycemia alters γδ T cell antitumor function. Vγ9Vδ2 T cell receptors recognize phosphorylated metabolites accumulating in cancer cells due to dysregulated mevalonate pathways or pharmacologic intervention (103). IL-17-secreting γδ T cells, relying on oxidative phosphorylation, often promote tumor progression, whereas IFN-γ–producing subsets, dependent on glycolysis, associate with tumor regression and favorable prognosis (104–106). Vγ9Vδ2 T cells from T2DM patients exhibit defects in synapse formation with target tumor cells and lytic granule polarization (21). The hyperglycemic diabetic environment induces pathological metabolic reprogramming, enhancing the Warburg effect (aerobic glycolysis) in Vγ9Vδ2 T cells, which suppresses AMPK activity and impedes lytic granule polarization and trafficking to the immunological synapse (21). The AMPK pathway also functions in tumor cells. Vγ9Vδ2 T cells recognize a cell surface complex containing Butyrophilin 2A1 (BTN2A1) and BTN3A1, overexpressed in malignancies (107). This complex can be activated by elevated levels of phosphoantigens in tumor cells. AMPK activation in tumor cells increases BTN2A1-BTN3A complex expression, enhancing Vγ9Vδ2 T cell-mediated tumor killing (107). Many antidiabetic drugs may modulate cancer risk (108). Metformin, as an AMPK agonist, may increase the expression of specific tumor cell surface proteins (BTN2A1 and BTN3A), potentially enhancing recognition by Vγ9Vδ2 T cells (21) (**Figure 3**). Various γδ T cell-based immunotherapeutic strategies, including *ex vivo* expanded allogeneic γδ T cells, γδ T cell infusion, and antibodies, are under clinical evaluation (103). This offers insight: deeper understanding of DM-γδ T cell interactions may enable analogous strategies to delay disease progression and improve quality of life in diabetic patients and those with complications.

### 4.3 Impaired wound healing

The skin harbors γδ T cells enriched in both the epidermis and dermis. These cells coordinate the complex interplay between keratinocytes and inflammatory cells by secreting growth factors and inflammatory mediators, thereby contributing to the regulation of epithelial homeostasis. Skin-resident γδ T cells exert protective functions and contribute critically to skin wound healing through multiple mechanisms, including costimulatory molecules, cytokine secretion, and chemokine production (109). This is highly relevant to understanding the mechanisms underlying chronic, non-healing wounds. Under diabetic conditions, hyperglycemia and obesity synergistically disrupt this repair network. Human chronic wound tissues contain more γδ T cells than normal tissues, but γδ T cell numbers are lower in chronic wounds of T2DM patients compared to non-diabetic chronic wound patients (26). Studies in diabetic mice reveal impaired IL-17 secretion by dermal Vγ4^+^ T cells due to reduced levels of IL-7, IL-23, and IL-1β; recruitment of Vγ4^+^ T cells is also diminished by attenuated CCL20/CCR6 chemokine signaling (109, 110). Notably, this research identified that impaired mTOR signaling in epidermal keratinocytes of diabetic mice leads to reduced IL-7 secretion, consequently impairing Vγ4^+^ T cell function. The mTOR inhibitor rapamycin can impair the wound-healing capacity of γδ T cells in mice (111). Similarly, in intact skin of streptozotocin (STZ)-induced diabetic rats, weakened mTOR pathway activation impairs the insulin like growth factor 1 (IGF-1)/IL-15 axis, disrupting keratinocyte-γδ T cell interactions and delaying wound closure (112, 113). Skin γδ T cells are activated via junctional adhesion molecule-like (JAML)-coxsackie and adenovirus receptor (CAR)costimulatory signals, inducing secretion of cytokines like IL-2 and TNF-α (114), thereby influencing wound healing processes involving outer-layer keratinocytes (26). IL-2 stimulation activates Jak1 and Jak3 in skin γδ T cells of mice (115), leading to phosphorylation of STAT5A and STAT5B, peaking at 30 minutes before rapidly declining (116). Under high-glucose conditions, IL-2 stimulation induces abnormally sustained STAT5A phosphorylation but fails to elicit STAT5B phosphorylation (116). Elevated TNF-α, associated with obesity and insulin resistance, suppresses the tissue-repair function of skin γδ T cells, whereas TNF-α blockade restores their epithelial responsiveness (116, 117). Collectively, hyperglycemia or obesity impairs skin γδ T cell proliferation and function via STAT5, aryl hydrocarbon receptor (AHR) signaling, mTOR, the IL-15–IGF-1 loop, and other pathways (22, 113, 116, 118) (**Table 4**). This renders γδ T cells unresponsive to epithelial damage and increases inflammation-associated gene expression.

**Table 4:** Immunologic mechanisms of diabetic wound healing failure in γδ T cells: dysregulated pathways and compromised γδ T cell-epithelial communication.

Molecular factors/pathways	Physiological role in wound progression or wound healing	Alterations in obesity/hyperglycemia/diabetes models	Outcome	Reference
AHR	Alleviates inflammation and maintains skin homeostasis by suppressing inflammatory genes and upregulating genes for cell morphology and ion homeostasis	↓ (AHR signaling in obesity)	↑ Expression of certain inflammatory genes (e.g., IFN-γ, GZMF, PDL-1); Epidermal γδ T cell accumulation and cytokine release, contributing to delayed wound repair	(22)
STAT5	STAT5A and STAT5B undergo rapid, transient phosphorylation	Prolonged STAT5A phosphorylation under hyperglycemia; Absence of STAT5B phosphorylation	↓ Proliferation of skin γδ T cells	(116)
IGF-1	Modulates keratinocyte secretion of IL-15, promoting wound closure	↓ (mTOR pathway impairment reduces IL-15 activation and IGF-1 levels; Basal keratinocyte layer in diabetic skin and foot ulcers lacks)	Negative impact on keratinocyte proliferation; Delayed wound closure	(109, 119–121)
Akt/mTOR	↑ (Total and phosphorylated protein levels of Akt, mTOR, p70S6K, 4E-BP1 and eIF4E in wound)	↓ (Total and phosphorylated protein levels of Akt, mTOR, p70S6K, 4E-BP1); ↑ (Phosphorylated eIF4E post-wounding)	↓ Number of dual-positive fibroblasts; Delayed re-epithelialization, impaired neovascularization, reduced synthesis of growth factors, collagen, and ECM	(113, 116)
TNF-α	Induces inflammation	↑ TNF-α levels	Skin γδ T cells become unresponsive to tissue damage	(122)
TGF-β1	Critical cytokine for wound repair	TGF-β1 fails to increase	Skin γδ T cells in obesity and metabolic disease fail to upregulate TGF-β1 at the wound edge	(116)

This table summarizes the physiological role of specific molecular factors and pathways in normal wound healing and their documented alterations in models of obesity, hyperglycemia, and diabetes. Arrows (↑ increase, ↓ decrease) indicate the direction of change in expression, phosphorylation, or activity of the specified factor in the diabetic state compared to healthy controls. The outcomes listed are the proposed cellular and physiological consequences of these molecular alterations, culminating in delayed wound repair. Key abbreviations: AHR, aryl hydrocarbon receptor; ECM, extracellular matrix; eIF4E, eukaryotic initiation factor 4E; GZMF, Granzyme F; IGF-1, insulin-like growth factor 1; 4E-BP1, eIF4E-binding protein 1; p70S6K, p70 S6 kinase.

Psoriasis, a chronic inflammatory skin disorder, is another skin-related comorbidity of DM. CCL20-mediated recruitment of γδ T cells via the CCR6 receptor, promoting increased IL-17 secretion, may be a key mechanism underlying delayed wound repair in diabetes. In psoriasis, enhanced CCR6 expression on skin γδ T cells of mice facilitates greater recruitment of dermal Vγ4^+^ T cells (123), which drive pathology through massive IL-17 secretion. IL-17, in turn, acts on keratinocytes to induce their proliferation (leading to epidermal hyperplasia) and the production of further pro-inflammatory cytokines and antimicrobial peptides, creating a self-amplifying inflammatory loop (124). This same pro-inflammatory pathway is hijacked in diabetic skin, contributing to impaired wound healing. CCL20 expression is elevated in the skin of T2DM patients (125), and dermal γδ T cells increase in the hind paws of 21-week-old db/db mice (126). The resulting IL-17-driven chronic inflammation disrupts the orderly process of wound repair by preventing the transition from the inflammatory to the proliferative phase. Chronic inflammation driven by pro-inflammatory cytokines from immune cells can stall diabetic wound repair (127). However, findings regarding γδ T cell numbers in wounds show discrepancies between studies. While Vγ4^+^ T cells play a significant role in chronic wound healing, both increasing and inhibiting IL-17A have been observed to accelerate wound healing in diabetic mice (109). This paradox may be explained by the concentration-dependent and temporal-specific role of IL-17; at early stages or low levels, it may aid in host defense and stem cell activation, whereas its persistent, high-level secretion is unequivocally pathogenic. Variations in wound pathology severity across non-healing models and limited sample sizes likely contribute to these conflicting results, highlighting the need for future research to explore whether skin γδ T cell loss or recruitment relates to other underlying factors. Furthermore, preventing chronic wounds may require a specific cytokine balance. These studies provide novel insights into the mechanisms of refractory wound formation in diabetic patients. As the autoimmunity mechanisms by which skin γδ T cells function in diabetic refractory wounds are further elucidated, fine-tuning cytokine levels holds promise for overcoming the therapeutic challenges of these wounds.

## 5 Immunomodulatory effects of antidiabetic drugs on γδ T cells

**Figure 4:**
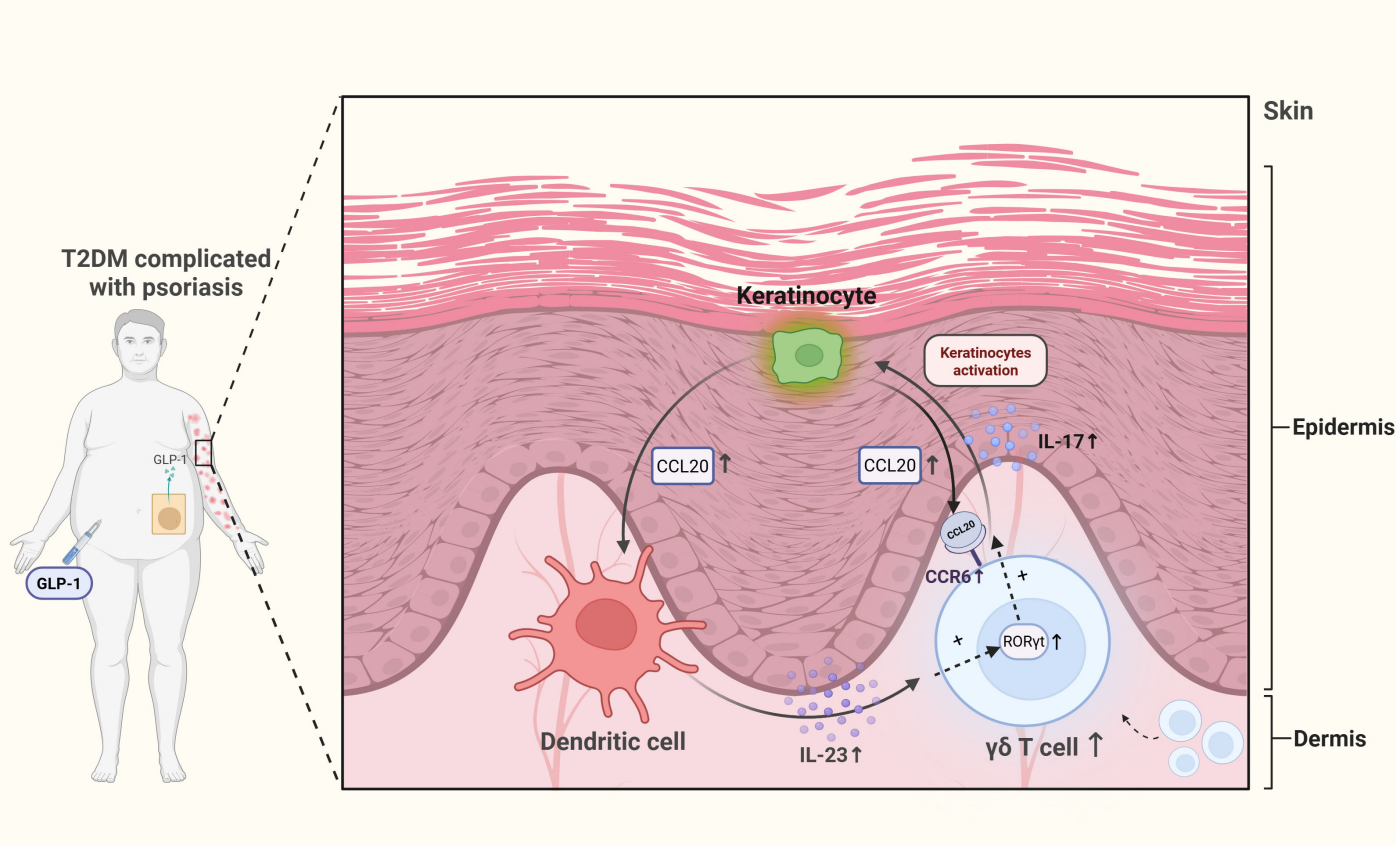
Dermal γδ T cell hyperactivation in T2DM-associated psoriasis and GLP-1RA intervention. Self-perpetuating inflammatory loop: Keratinocyte-derived CCL20 recruits dermal γδ T cells, whose IL-17 secretion further stimulates keratinocytes to upregulate CCL20, by binding to CCR6 receptors. Cytokine and metabolic amplifiers: DC-derived IL-23 polarizes γδ T cells toward a RORγt^+^IL-17^+^ phenotype, while T2DM-associated hyperglycemia synergistically enhances both CCL20 production and IL-17 output. GLP-1 receptor agonists (GLP-1RAs) interrupt this cycle by modulating metabolic and inflammatory pathways.

Current research on the mechanisms of antidiabetic drugs is shifting from solely metabolic regulation towards dual immunometabolic perspectives. As pivotal bridging cells, γδ T cells represent a functional target for multiple therapeutic approaches (**Table 5**). 1α,25(OH)_2_D_3_ is the active form of Vitamin D, which is also known as Calcitriol. It improves systemic insulin resistance by suppressing inflammatory responses in γδ T cells. Acting via the vitamin D receptor (VDR), 1α,25(OH)_2_D_3_ promotes FBP1 expression, inhibits glycolysis in human Vδ2 T cells, and consequently reduces pro-inflammatory cytokine production (105). Mucosal insulin administration, inducing regulatory CD8^+^ γδ T cells, represents another potential strategy for preventing human T1DM (57). PBMCs from T2DM patients treated with saxagliptin, a dipeptiyl peptidase 4 (DPP-4) inhibitor, exhibit reduced levels of IL-23, JAK2, STAT3, and RORγt (81). As RORγt is a key transcription factor for γδ T cells, these cells may be modulated by DPP-4 inhibitors, potentially contributing to improvements in glucose homeostasis and metabolic control in T2DM. Additionally, psoriasis exhibits a significant association with DM (131, 132). A prospective case study found that applying GLP-1 receptor agonists (GLP-1 RAs) in T2DM patients with comorbid psoriasis reduced dermal γδ T cell numbers and IL-17 production, correlating with improved clinical severity of psoriasis (128). Given that psoriasis involves elevated γδ T cells and IL-17 production, these findings suggest that GLP-1 RAs may offer benefits for psoriasis management alongside glucose-lowering and weight-loss effects. This finding provides a novel rationale for selecting glucose-lowering regimens in diabetic patients, specifically favoring GLP-1 RAs for T2DM patients with concurrent psoriasis (**Figure 4**). Further research reveals abundant GLP-1 receptor expression on murine small intestinal γδ T cells (129, 130), suggesting that GLP-1 RAs may directly modulate γδ T cells and influence autoimmune responses, potentially delaying diabetes onset and progression. However, specific mechanistic evidence for this remains lacking and warrants investigation.

**Table 5:** Dual metabolic-immune targeting in DM and comorbidities: γδ T cell-directed therapeutic strategies.

Therapeutic agent	Target/context	Effect on γδ T cells	Key mechanism(s)	Therapeutic significance	Reference
1α,25(OH)_2_D_3_	T2DM Insulin Resistance	Suppresses inflammatory response	Activates VDR receptor → ↑ FBP1 expression; Inhibits glycolysis → ↓ Pro-inflammatory cytokine production	Improves systemic inflammation and insulin sensitivity	(105)
GLP-1 Receptor Agonists	T2DM patients with comorbid psoriasis	↓ Dermal γδ T cell numbers	Reduces IL-17 (key pathogenic factor in psoriasis) production of γδ T cells	Ameliorates skin inflammation and metabolic parameters	(128)
	Murine intestinal γδ T cells	? (No direct evidence)	High GLP-1 receptor expression → Potential for direct modulation	Novel immunometabolic regulatory target	(129, 130)
Aerosol Insulin	T1DM prevention (Animal/Clinical exploration)	Induces expansion of regulatory CD8^+^ γδ T cells	Promotes immune tolerance/suppresses autoimmunity: ↑ IL-10-secreting CD8^+^ γδ T cells in pancreatic lymph nodes	Potential prevention of T1DM onset	(57)
DPP-4 Inhibitors (e.g., Saxagliptin)	T2DM patients	? (No direct evidence)	Suppresses key pathway (JAK2/STAT3/RORγt) for Th17/γδT17 cell differentiation → ↓ IL-17	Reduces inflammatory state; Potential improvement in islet function	(81)

This table summarizes the immunomodulatory effects of various diabetic therapeutics on γδ T cell populations, their proposed mechanisms of action, and their therapeutic significance. A question mark (?) indicates that direct evidence for an effect on γδ T cells is currently lacking, and the proposed mechanism is inferred from related pathways or cell types. Key abbreviations: DPP-4, dipeptidyl peptidase-4; FBP1, fructose-1,6-bisphosphatase 1; GLP-1, glucagon-like peptide-1; JAK2, Janus kinase 2; RORγt, retinoic acid receptor-related orphan receptor gamma-t; STAT3, signal transducer and activator of transcription 3; T1DM, Type 1 diabetes mellitus; T2DM, Type 2 diabetes mellitus; Th17, T helper 17 cell; VDR, vitamin D receptor.

↑ (Upward arrow): Indicates upregulation, increase, or enhancement. ↓ (Downward arrow): Indicates downregulation, decrease, or suppression. → (Rightward arrow): Denotes leads to, results in, or promotes a subsequent biological effect or outcome.

Therapeutically harnessing γδ T cells requires precision targeting of autoimmune-specific pathways. In T1DM, agents targeting IL-17 and related pathways, such as ustekinumab and ixekizumab, are currently in Phase II/III recruiting stage (39, 133, 134). Beyond monoclonal antibodies, safe and effective oral small molecule drugs (SMDs) targeting molecules like RORγt (39) hold future clinical promise, although such agents have not yet advanced to clinical studies. While IL-17 and RORγt are closely linked to γδ T cells, they are not γδ T cell-specific targets, as other immune cells also utilize these pathways. The potential of γδ T cells as early warning biomarkers or immunomodulatory targets remains underexplored, particularly for high-risk diabetes populations. Given that up to 50% of individuals with prediabetes progress to diabetes within 5 years (135), strategies to delay or reverse this progression are crucial. Significant potential also exists for novel γδ T cell-targeted drug development. Local insulin injection accelerates diabetic foot ulcer healing by stimulating AKT and ERK pathways (136). As γδ T cells recognize certain insulin antigens and their TCR activation involves MAPK pathways, investigating whether insulin promotes γδ T cell function to aid wound healing in diabetes presents a promising avenue for future research. Collectively, substantial gaps remain in developing clinically applicable γδ T cell-based strategies for preventing or treating diabetes, demanding further exploration.

## 6 Discussion

This review comprehensively summarizes the dual regulatory roles of γδ T cells in DM and its complications. On the one hand, γδ T cells contribute to diabetes pathogenesis through the secretion of effector molecules like IL-17, with functions exhibiting subset-specific characteristics. On the other hand, the diabetic pathological milieu reciprocally impairs γδ T cell functions in anti-infection, antitumor autoimmunity, and tissue repair.

γδ T cells may also modulate regulatory T cells (Tregs) or other immune cells, thereby potentially alleviating certain pathological processes. In AT of mice, both γδ T cells and Tregs increase with age, with γδ T cells and their secretion of IL-17 providing a conditioning signal for Treg accumulation (69). During influenza virus clearance or AT thermoregulation, IL-17-secreting γδ T cells may promote Treg accumulation via IL-33 upregulation (69, 137). Studies have identified impaired function or reduced numbers of Tregs in both T1DM and T2DM patients (138, 139). Tregs can also improve insulin resistance by suppressing the activity of Th1, Th2, and Th17 cells (138). Therefore, γδ T cell–mediated promotion of Treg accumulation and functional restoration via IL-17A secretion may represent a potential strategy for future diabetes immunotherapy. In addition to their direct antitumor functions, γδ T cells also exert indirect antitumor effects by modulating αβ T cell activity (140).

Research on the role of γδ T cells in DM still faces significant limitations that require resolution. Firstly, current investigations inadequately characterize the functions of human γδ T cell subsets; findings derived from mouse subsets cannot be directly extrapolated to humans. The specific role of the tissue-resident Vδ1 subset in diabetes and its complications remains unclear. This ambiguity stems partly from the difficulty in obtaining samples, as Vδ1 cells primarily reside in the skin and MALT. Utilizing organoid models to simulate tissue-resident subset function may offer a solution. Furthermore, the Vδ1 subset is scarce in peripheral blood and does not recognize phosphoantigens, presenting challenges for direct ex vivo expansion and culture in research settings. Secondly, the precise role of γδ T cells across different stages of diabetes progression remains poorly defined. The dynamic changes and underlying mechanisms of γδ T cells, from the stage of impaired glucose tolerance in prediabetes to the onset of acute or chronic diabetic complications, remain poorly understood.

While these unresolved complexities pose challenges, therapeutically targeting the metabolic susceptibility and TCR-dependent reprogramming of γδ T cells may help translate mechanistic insights into future clinical applications for DM and its comorbidities. Elucidating the context-dependent duality of γδ T cells in DM not only deciphers the immunometabolic crosstalk at the cellular frontier but also unlocks precision-targeted therapies.

## Glossary

DM: iabetes mellitus
T1DM: Type 1 diabetes mellitus
T2DM: Type 2 diabetes mellitus
AT: Adipose tissue
IR: Insulin resistance
MALT: Mucosa-associated lymphoid tissue
MHC: Major histocompatibility complex
HMBPP: (E)-4-hydroxy-3-methyl-but-2-enyl pyrophosphate
IL: Interleukin
IFN-γ: Interferon-gamma
TCR: T cell receptor
DETC: Dendritic epidermal T cell
DC: Dendritic cell
PAMP: Pathogen-associated molecular pattern
PRR: Pattern-recognition receptor
ERK/MAPK: Extracellular signal–regulated kinase/mitogen-activated protein kinase
NK: Natural killer
NR4A: Nuclear receptor subfamily 4 group A
GDM: Gestational diabetes mellitus
NOD: Non-obese diabetic (mouse)
ICA: Islet cell antibody
PBMCs: Peripheral blood mononuclear cells
DKD: Diabetic kidney disease
mTOR: Mechanistic target of rapamycin
AMPK: AMP-activated protein kinase
BTN: Butyrophilin
BTN2A1: Butyrophilin subfamily 2 member A1
BTN3A: Butyrophilin subfamily 3 member A
STAT: Signal transducer and activator of transcription
RORγt: Retinoic acid receptor-related orphan receptor gamma t
AHR: Aryl hydrocarbon receptor
IGF-1: Insulin-like growth factor 1
HF diet: High-fat diet
GLUT4: Glucose transporter type 4
IRS1: Insulin receptor substrate 1
